# The role of fine-needle aspiration in the thyroid nodules of elderly patients

**DOI:** 10.18632/oncotarget.7643

**Published:** 2016-02-23

**Authors:** Esther Diana Rossi, Tommaso Bizzarro, Maurizio Martini, Patrizia Straccia, Celestino Pio Lombardi, Alfredo Pontecorvi, Luigi Maria Larocca, Guido Fadda

**Affiliations:** ^1^ Division of Anatomic Pathology and Histology, Università Cattolica del Sacro Cuore, “Agostino Gemelli” School of Medicine, Rome, Italy; ^2^ Division of Endocrine Surgery, Università Cattolica del Sacro Cuore, “Agostino Gemelli” School of Medicine, Rome, Italy; ^3^ Division of Endocrinology, Università Cattolica del Sacro Cuore, “Agostino Gemelli” School of Medicine, Rome, Italy

**Keywords:** elderly patients, thyroid lesions, malignancies, liquid based cytology, immunocytochemistry, Gerotarget

## Abstract

We assess the role of thyroid fine needle aspiration cytology(FNAC) in our series of elderly patients. The growing subset of people aged older than 70 years has shown an increased incidence of thyroid diseases which need to be studied in order to reduce the percentage of surgical treatments in patients with higher likelihood of co-morbidities and associated life risk. We compared Follicular/Indeterminate Neoplasms(FN) and suspicious of malignancy(SM) with pediatric and adult cohorts. We discussed the role of immunocytochemistry-ICC to refine diagnoses. Four hundred and eighty out of 3539FNACs(13.5%) in elderly patients, were surgical followed-up. They included: 35Inadequate, 188benign(BL), 164FN/AUS, 49SM and 44positive for malignancy (PM). All PM and 95.7%BL were histological confirmed. The malignant rate was 24.3% mostly diagnosed as papillary thyroid carcinomas. An ICC panel (HBME-1 and Galectin-3) was carried out on liquid based cytology (LBC) and performed on FN/AUS, SM and PM. We found concordant positive ICC in 69.3%malignancies and concordant negative ICC in 97.6%benign follicular adenomas. Among FNs, 42.9%malignant histologic cases had concordant positivity whilst 97.4%benign histology had negative panel. Thyroid FNAC shows high feasibility in elderly patients. ICC helps in reducing the number of useless thyroidectomies and providing a more adequate clinical and/or surgical selection in elderly patients.

## INTRODUCTION

Thyroid nodules are a common finding with up to 50% of the patients with at least one thyroid nodule characterized by an average and estimate risk of malignancy at about 5% [1-2]. Whereas the majority of the studies deal with the prevalence of thyroid nodules in adult population, they do not specify and investigate thyroid diseases among the so called elderly population including patients from 70 to 95 y/o [3-5]. Until now the role of patients' age on thyroid lesions has shown contradictory results [3-5]. Recently Kwong et al demonstrated 1.6% increased multinodular thyroids but on the other hand the reduction in the percentage of cancers [3]. Whilst no single clinical and radiological test has proven to show 100% sensitivity and specificity, fine-needle aspiration cytology (FNAC) is likely to be considered as the most important and primary diagnostic tool for the evaluation and diagnosis of thyroid lesions regardless of patient age. In fact it has worldwide application because of its simplicity, safety, and cost-effectiveness [6-13]. However, all the different classification systems currently in use for reporting thyroid cytopathology do not include any specific criteria for the elderly subset of patients affected more than other categories of the different impact of the clinical and/or surgical management [14-16]. Nevertheless, as observed in general population, the detection of indeterminate or follicular proliferations on a cytological smear in elderly patients implies the same difficulties in attributing the correct diagnostic category and following the proper management [3, 11-13].

The issues attributed to the well-known limits of the morphological results, mostly within the “gray zone” of the follicular proliferations, may be overcome by the application of ancillary techniques including both immunocytochemistry (ICC) and molecular analysis [1-2, 17-19]. In fact as demonstrated by several authors, these tools can significantly empower the morphological diagnosis and prognosis of FNs allowing a more accurate prediction of the nature of the lesion. In the present study we conducted a retrospective analysis of all our consecutive elderly patients (aged 70-95 y/o) with thyroid nodules between 2000 and 2013. Additionally we provided the comparative evaluation of both pediatric and adult cohorts in the same reference period. Furthermore, based on our previous experiences with ICC in adult and pediatric thyroid series [1-2,20], we evaluated whether the application of the same immunopanel comprised of Hector Battifora mesothelial cell-1 (HBME-1) and Galectin-3 might avoid unnecessary surgery in these elderly patients frequently affected by co-morbidities and disease burden.

## RESULTS

### Study population

We performed a retrospective and computerized search of all consecutive elderly cytological cases recorded in the files of the Division of Anatomic Pathology and Histology of the Catholic University of the Sacred Heart, Agostino Gemelli Hospital, between January 2000 and December 2013. Out of 30.144 thyroid FNACs, we obtained a cohort of 3539 cytological cases of patients aged 70-95 y/o. Four hundred and eighty patients had surgical follow-up.

### Clinical-pathological results

A total of 480 elderly thyroid FNACs (aged 70-95 y/o) with surgical follow-up were recorded during the study period (January 2000-December 2013) from a total of 33489 thyroid FNAC cases (including 220 pediatric, 26385 adult and 3539 elderly populations). All the specimens were processed using LBC method. These 480 cyto-histological cases resulted in 129 (26.8%) male and 351 (73.2%) female patients with a median age of 79 y/o. Concerning the size, the cases smaller than 2 cm doubled those larger than 2 cm (336 cases vs 144). We esteemed also the distribution of cases for each cytological category including 35 TIR1/IN (7.3%), 188 TIR2-BL (39.2%), 164 TIR3-FN/AUS (34.2%), 49 TIR4/SM (10.2%) and 44 TIR5/PM (9.1%). The results of the histological diagnoses, reported in the same Table 1, confirmed the higher percentage of benign lesions (363-75.6% cases) versus malignancies (117-24.3%). Specifically the most frequent benign entity was goiter whilst PTC was the most common among malignancies. We also analyzed the T and N stages for each malignant histological sample as summarized in Table 1. The data univocally demonstrated the prevalence of TI-TII and pN0 regardless in the malignant histology.

**Table 1:** Clinico-pathological features of the series including 480 elderly patients with surgical follow-up

Clinical Features	No. of cases (%)
Sex, n	
Male	129 (26.8%)
Female	351 (73.2%)
Age range (median)	
70-95 y.o.	480 (79 y.o.)
Size, n	
< 2 cm	336 (70%)
> 2 cm	144 (30%)
Cytology, n	
TIR1	35 (7.3%)
TIR2	188 (39.2%)
TIR3	164 (34.2%)
TIR4	49 (10.2%)
TIR5	44 (9.1%)
Histology, n	
Goiter	213 (44.5%)
Adenoma	150 (31.3%)
PTC	59 (12.3%)
FVPTC	37 (7.7%)
FTC	5 (1%)
MTC	6 (1.2%)
HCC	6 (1.2%)
ATC	3 (0.6%)
Sarcoma	1 (0.2%)
T- Stage, n	
TI-TII	99 (84.6%)
TIII-TIV	18 (15.4%)
N-Stage, n	
pNO	106 (90.6%)
pN1	11 (9.4%)

Legend: PTC:Papillary thyroid carcinoma; FVPTC: Follicular variant of PTC; FTC: Follicular carcinoma; MTC: Medullary thyroid carcinoma; HCC: Hurthle cell carcinoma; ATC: Anaplastic Thyroid carcinoma. N0: negative lymph nodes; Pn1: positive lymph nodes.

The specific cyto-histological correlation for these cases is depicted in Table 2. Specifically the 35 TIR1/IN with surgical pathology follow-up included 22 goiters, 9 FAs, 2 PTCs and 2 FVPCs. Overall 180 of 188 (95.7%) TIR2/BL resulted in a benign histology (149 goiters and 31 FAs) and 8 (4.3%) in malignant histological diagnoses (3 PTC, 3FVPC, 1 FC, 1MTC). A total of 164 TIR3-FN/AUS underwent surgical excision; 133 had benign (39 goiters and 94 FAs) and 31 had malignant (9PTCs, 14FVPCs 3FC, 5 HCC) follow-up. Surgical pathology follow-up was available in 49 cases diagnosed as TIR4-SM; 19 were diagnosed as benign (3 goiters and 16 FAs) and 30 as malignant (13 PTC, 13 FVPC, 1FC, 1MTC, 1HCC, 1 Sarcoma) on histopathologic interpretation. A total of 44 cases diagnosed as TIR5/PM on FNAC underwent surgical excisions; all were found to be malignant (32 PTC, 5FVPC, 4MTC, 3ATC). Table 2 also shows the different histotypes among the cytological categories. We found a total of 59 papillary thyroid cancers (PTC) with the majority of the cases (76%) within the SM (13 cases) and PM (32 cases) categories; 37 follicular variant of PTC (FVPC) with a strong prevalence (73%) between the FN/AUS (14 cases) and the SM (13 cases) categories; 5 follicular thyroid cancers (FTC) and 6 Hurtle cells carcinomas (HCC) almost all in the FN/AUS category, whereas most of the 6 medullary carcinoma (MTC) and all the 3 anaplastic carcinoma (ATC) were found in the PM category. One SM resulted in a misdiagnosis of sarcoma. The FVPCs include 20 encapsulated and 17 infiltrative carcinomas.

Furthermore, in the same Table 2 we worked out also the relative risk of malignancy for each category. Concerning the malignancies reported in the different categories, we found 4 of them out of 35 Non-diagnostic samples (11,4%), 8 out of 188 BL (4,3%), 31 out of 164 FN/AUSs (18,9%), 29 out of 48 SMs (60,4%), and all 44 PM cases (100%).

**Table 2:** Cyto-histological correlation in the series

CYTOLOGY (n)	HISTOLOGY (n)											
	Goiter	FA	BENIGN CASES	PTC	FVPC	FTC	MTC	HCC	ATC	Sarcoma	MALIGNANT CASES	ROM
TIR1 (35)	22	9	31	2	2	-	-	-	-	-	4	11.4%
TIR2 (188)	149	31	180	3	3	1	1	-	-	-	8	4.3%
TIR3 (164)	39	94	133	9	14	3	-	5	-	-	31	18.9%
TIR4 (49)	3	16	19	13	13	1	1	1	-	1	30	61.2%
TIR5 (44)	-	-	0	32	5	-	4	-	3	-	44	100.0%
Tot. (480)	213	150	363	59	37	5	6	6	3	1	117	24.4%

FA: Follicular adenoma; PTC: Papillary thyroid carcinoma; FVPTC: Follicular variant of PTC; FTC: Follicular carcinoma; MTC: Medullary thyroid carcinoma; HCC: Hurthle cell carcinoma; ATC: Anaplastic Thyroid carcinoma. pN0: negative lymph nodes; pN1: positive lymph nodes; ROM: risk of malignancy.

### Comparative analysis of different cohorts of patients

**Figure 1:**
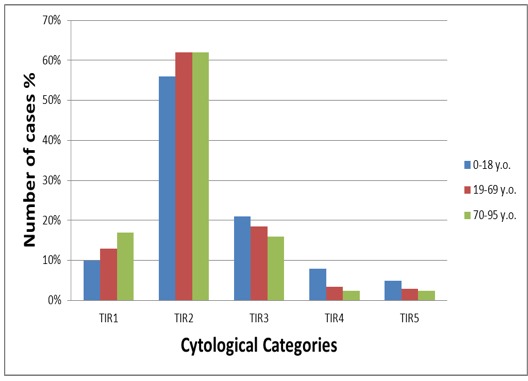
Graphic representation of the distribution of the three cohorts of patients set up according to age

In Table 3 and Figure 1 we compared the distribution of the entire series of thyroid lesions among three cohorts of patients (specifically 0-18 y/o, 19-69 y/o and 70-95 y/o) set up according to their age. As depicted in the table and Figure, the categories of TIR1/IN, TIR2/BL and TIR3-FN/AUS remained stable among the three cohorts whilst TIR4/SM in elderly was threefold lower than in the pediatric category and TIR5/PM halved in elderly patients (Table 3 and Figure 1).

**Table 3:** Comparative analysis among the different cohorts of patients

Cytological Categories	Distribution of cases into age groups, n (%)		
	0-18 y.o.	19-69 y.o.	70-95 y.o.
TIR1	22 (10%)	3400 (13%)	613 (17%)
TIR2	123 (56%)	16382 (62%)	2184 (62%)
TIR3	47 (21%)	4861 (18,5%)	577 (16%)
TIR4	17 (8%)	913 (3,5%)	84 (2,5%)
TIR5	11 (5%)	829 (3%)	81 (2,5%)
TOT	220	26385	3539

The distribution of categories is accordingly to those reported in methods.

### ICC yields

In agreement with our previous studies (1-2, 20), we gagged the application of an immunopanel made up of HBME-1 and Galectin-3 in all the 257 cytological samples diagnosed as FN/AUS, SM and PM (Table 4a). We did not perform ICC on the BL category due to the clear and unequivocal benign morphological features as also confirmed by 95.7% benign histology. In the light of the lack of adequate cellular component, we did not carry out ICC on our 35 TIR1. Table 4a estimated the correlation of the histological diagnoses with the ICC panel expression. The ICC analysis was considered informative only for the cases with a positive or negative concordant immunopanel based on the evidence that any single immunomarker positivity does not provide unequivocal diagnostic results. For this reason 70 cases with discordant positivity (only 1 immunomarker expression) were considered as a negative panel.

**Table 4a:** Cyto-histological correlation for the ICC panel (FN/AUS, SM and PM)

ICC	Histological Diagnoses, n (%)		
	Benign	Malignant	Overall
HBME1 - / Galectin3 -*	81 (97.6%)	2 (2.4%)	83
HBME1 + / Galectin3 +*	32 (30.7%)	72 (69.3%)	104
HBME1 + / Galectin3 -	16 (44.4%)	20 (55.6%)	36
HBME1 - / Galectin3 +	23 (67.6%)	32.4%)	34

Legend: ICC: Immunocytochemistry.

* *p*-value <0.005

The concordant immunopanel resulted in 83/187 (44.3%) negative and 104/187 (55.7%) positive with statistical significance (*p* < 0.05). Eighty-one out of 83 (97.6%) cases with concordant negative panel were histological follicular adenomas whereas we had 2 false negative concordant immunopanel (belonging to the FN/AUS category) which were histological malignant (1PTC and 1FVPC) (*p* < 0.05). All our 59 papillary carcinomas and 13 FVPC resulted positive for the complete immunopanel nevertheless we had 32 cases with concordant immunopanel (mostly belonging to the FN/AUS category) resulted in a histological benign outcome (namely follicular adenoma). Next, we analyzed the 70 cases with discordant panel. Twenty out of 36 (55.6%) cases showing only HBME-1 positivity were associated with a malignant histology whilst the discordant panel with Galectin-3 expression was found in only 32.4% of the malignant outcome (Table 4a).

Table 4b describes the evaluation of ICC for the category of the 164 FN/AUS cases. The concordant immunopanel was positive in 21 (42.9%) malignant cases with 57.1% false positive benign histology (follicular adenomas) showing statistical significance (*p* < 0.05). However the negative concordant panel was confirmed to be associated with a benign histology even when we considered the FN/AUS category alone (97.4%, *p* < 0.05). Based on the application of our immunopanel we obtained 93.7% sensitivity, 71.7% specificity, 69.2% positive predictive value (PPV), 97.6% negative predictive value (NPV) with the 81.8% diagnostic accuracy, whereas if we consider specifically the indeterminate (FN/AUS) category our figures resulted in 91.3% sensitivity, 72.8% specificity, 42.9% PPV, 97.4% NPV with the 76.2% of diagnostic accuracy.

**Table 4b:** Cyto-histological correlation for the ICC panel (FN/AUS)

ICC	Histological Diagnoses, n (%)		
	Benign	Malignant	Overall
HBME1 - / Galectin3 -*	75 (97.4%)	2 (2.6%)	77
HBME1 + / Galectin3 +*	28 (57.1%)	21 (42.9%)	49
HBME1 + / Galectin3 -	13 (76.4%)	4 (23.6%)	17
HBME1 - / Galectin3 +	17 (81%)	(19%)	21

Legend: ICC: Immunocytochemistry.

* *p*-value <0.005

## DISCUSSION

Our study deals with the analysis of the prevalence of thyroid nodules and their risk of malignancy in a series including elderly patients during a 13 year span period. A conclusive analysis of the thyroid nodules in elderly patients has been not univocally defined due to the scant and contradictory data by literature [3-5].

Nonetheless, few recent papers assessed that the prevalence of thyroid nodule in general population ranges between 4% by palpation to 67% by ultrasonography with a steadily growing number of thyroid nodules in patient older than 65y/o [3-5]. Other studies, mostly evaluating asymptomatic elderly patients, confirmed the tendency toward an increased prevalence of thyroid nodules in patients over 65 years of age, mostly diagnosed as goiter or benign lesions but accompanied also by a peak of thyroid cancer mostly represented by well differentiated thyroid carcinoma with also frequent recurrences [25-26].

Since these elderly patients face with higher incidence of trauma, neoplasms and other diseases than younger population, the correct cytological diagnosis of thyroid nodules may have significant implication mainly for the possible complications induced during surgical procedures. In this perspective the need of a correct cytological diagnosis is mandatory for maximizing the benefits of a medical or radio-iodine therapy rather than the risk of an unnecessary surgical management. Undoubtedly, FNAC, leading to a correct diagnosis in more than 70% of the cases in general population, represents the gold mark for achieving the appropriate management and reducing the number of benign nodules undergoing thyroid surgery [1-3, 11-14].

In agreement with other series from literature, we noticed that female population doubled male patients but in contrast with Knowg et al, we found that nodules smaller than 2 cm were twice those larger than 2 cm [3]. However, in agreement with other papers we did find that clinical characteristics, such as gender and age may significantly impact the risk of malignancy [3-5]. Moreover, our data confirmed the higher prevalence of benign lesions versus malignancies (75.6% vs 24.4%) namely characterized by goiter and PTC as the most frequent and specific diagnoses. However in the analysis of the data for each cytological category, we found that BLs represented only 39.2% of the entire surgical cohort. This figure is likely to be affected by the surgical bias as long as we acknowledged that BLs of the entire elderly population were 62%. Such discrepancy in the two groups of BL nodules (surgical vs entire elderly FNAC) suggested that the majority of them have been correctly followed-up and that our series includes those patients who underwent FNAC and surgery because of the detection of suspicious or/and symptomatic nodular lesions. Hence, in our cohort a total of 34.2% were FN/AUS demonstrating high likelihood of benign histology (85%) with only a small proportion of malignant outcome. Importantly, the risk of malignancy for each category was likely to be in agreement with the average threshold for the corresponding categories suggested by both the Italian and Bethesda classification systems as summarized by 4.3% in the BLs, 100% in the PMs and mostly by the value of 18.9% (lower than 20%) in the FNs [14, 16]. In order to describe the prevalence of thyroid lesions and diagnoses in elderly patients, we designed and investigated a comparative analysis of the different thyroid categories with the two cohorts of patients including childhood and adulthood. In this perspective, as underscored in Table 3, we did not find significant differences throughout all age cohorts especially in the BLs whilst only a slight reduction for the TIR3-FN/AUSs in elderly patients which may suggest the attempt of reducing the burden of the gray zone in these patients. Additionally, the comparative analysis of the three aged cohorts for the SMs and PMs documented a decrease in the malignant prevalence that such categories provided in elderly patients. The expected evidence that TIR5 in pediatric population doubled the value in the elderly group cannot refute the argument, also well-known by literature, that pediatric population would provide higher prevalence of malignancies with also more aggressive features [20]. However according to Knowg et al and other authors, a second peak of malignancy is also assessed in patients older than 65 y/o and they were likely to be aggressive and with poorly differentiated histotypes [3-7]. In fact the data summed up in our series confirmed a higher number of anaplastic and Hurthle cell carcinomas which are likely to be linked with a poorer prognosis and response to therapies.

Similar results were reported by Belfiore et al finding the highest rate of malignancies in both older patients (ages 71-80) and in younger than 20 years whilst Bessey et al, in contrast with the latter results, justified their yields with the selection of cold versus other types of thyroid nodules [5, 9]. However, it stands for reasons that FN/AUSs represent a challenge regardless of the patient age [10-14]. Hence, the majority of authors assess that the cytological features of both “positive for malignancy” and “benign lesions” are unequivocally identified by cytopathologists (experienced or not) whilst the categories of FN/AUS and SM depicted the so-called “ grey zone” including both benign and malignant outcome with different management and treatment especially in elderly patients [11-13]. Despite the lower incidence of FN/AUSs in the entire cohort of 3539 elderly patients, we observed a higher indeterminate rate in the surgical series (34.2%) which may be attributed to either a cautious approach or the desire not to lose them in the follow-up.

As found in another publications by our group, we performed ICC in all our FN/AUSs and SMs cases in order to reduce the surgical risks in elderly patients with advanced age and co-morbidities [1, 2, 20].

As previously deemed, we highlighted the valuable use of LBC as a reliable preparation minimizing some of the hitches encountered when ancillary techniques (e.g. molecular markers and ICC) are carried out on conventional slides [27-31].

In fact, the good results experienced the application of an immunopanel made up of HBME-1 and Galectin-3 led us to study all the 257 cases diagnosed as FN/AUS, SM and PM with the same ICC panel [1, 2, 20]. Arguments put forward in favor of the proposal use of multiple immunomarkers are associated within the evidence that none of the immunomarkers alone shows a diagnostic accuracy sufficient for being used as single antibody in the diagnosis of malignant thyroid neoplasm. When we considered the whole group of 257 cases, the useful role of a concordant negative immunopanel was confirmed in 97.6% of the histological follicular adenoma whilst only 69.3% of the cases with positive panel were associated with malignant histology. However FN/AUSs alone provided high negative predictive value (97.4%) and low (42.9%) positive predictive values confirming the high number of benign cases with a concordant negative panel as well as the high percentage of false positive cases with concordant ICC panel. Moreover, in contrast with our previous yields in both the adult and childhood series [1-2, 20-32], the analysis of ICC depicted a different scenario underscoring that the positivity of single immunomarkers was expressed in a series of 31 cases with malignant outcome. In the present paper we have found 70 cases with discordant panel (defined by the expression of only 1 marker) with 44.3% of them resulted in histological malignancy. However, other authors, such as Cochand-Priollet et al, have found 6 false positive LBC thyroid lesions without any false negative case applying an ICC panel of HBME-1 and Cytokeratin 19 [31]. However given the scant stored material and the false positive cases previously detected with the use of Cytokeratin 19, we did not perform any additional immunomarker even in cases with discordant panel. Despite the fact that several authors emphasized that none of the immunomarkers alone shows a diagnostic accuracy sufficient for being used as single antibody useful for the diagnosis of malignant thyroid neoplasms, the detailed evaluation of our ICC panel in FN/AUS enforced the malignant role of HBME-1 and Galectin-3 also separately. In fact, our yields suggested that even the positivity of a single immunomarker should be considered as a clue of malignant outcome encouraging for instance the analysis of somatic mutations as an additional diagnostic approach in cases with discordant panel. As long as the present study was a retrospective analysis we did not have sufficient stored material on cytological slides for these additional tests.

In summary, a valid strategy may include an accurate morphological evaluation accompanied by the assessment of the expression of the immunopanel (made up of HBME-1 and Galectin-3 or a single immunomarker are likely to identify low risk nodules and enable a better selection of surgical patients with a reduction of useless and dangerous thyroidectomies.

Furthermore in the FN and/or SM cases with contradictory ICC, the application of molecular analysis may support morphology for achieving a correct definitive diagnosis and treatment for throughout aged cohorts.

## MATERIALS AND METHODS

### Fine needle aspiration

All the nodules were evaluated under ultrasonographic guidance (US) mostly by surgeons and endocrinologists. All the cases were processed using the ThinPrep liquid-based cytology (LBC) method (Hologic Inc, Marlborough, Massachusetts-USA). All FNAs (usually 2 passes for each lesion) were performed with 25-gauge to 27-gauge needles without rapid adequacy assessment of the material. The nodule ranged in size from 0.5 cm to 6 cm. All the sub-centimeter lesions were discovered during routine ultrasound thyroid examination performed in the study institution.

### FNAC preparation

With regard to the use of LBC, all the patients were appropriately informed about the FNA method and all provided written informed consent. Our study followed the tenants of the Declaration of Helsinki and we received the internal ethical approval for the study. The aspirated material was fixed with the hemolytic and preservative CytoLyt solution (Cytyc Corporation, Marlborough, Mass) after rinsing the needle in this solution. The cells were spun at 1500 revolutions per minute and the sediment was then transferred to PreservCyt solution (Hologic Inc, Marlborough, Mass) to be processed with the T2000 and T5000 automated processor (Hologic Inc, Marlborough, Mass) according to the manufacturer's suggestions. The resulting slide was fixed in 95% methanol and stained with the Papanicolaou method, whereas the remaining material was stored in PreservCyt solution at room temperature for 3 to 4 months to be used for eventual additional investigations such as ICC and molecular analysis. These ancillary techniques can be performed when the remaining material is at approximately 2 ml eluted in 5 ml of PreservCyt solution.

### FNAC classification system

The cytological cases were classified according to the Italian Working Group SIAPEC-IAP (Società Italiana di Anatomia Pathologica e Citopatologia Diagnostica-International Academy of Pathology) classification [16]. The above-mentioned categories are defined as follows: TIR1 - inadequate or cystic-hemorrhagic sample-IN; TIR2 - non-neoplastic lesion (BL); TIR3 - follicular/indeterminate neoplasm including atypical cells of indeterminate significance (FN/AUS); TIR4 - suspicious for malignancy (SM); and TIR5 - positive for malignancy (PM). Although the cytological cases were classified according to the Italian Working Group SIAPEC-IAP classification, the majority of categories overlapped with the diagnoses adopted by the Bethesda System for Reporting Thyroid Cytopathology [14]. In fact the cytological diagnoses of benign lesions, suspicious for malignancy and positive for malignancy share identical features in both systems; indeed the TIR3 corresponds to the FN and AUS/FLUS (Atypia of undetermined significance-AUS/Follicular lesion of undetermined significance-FLUS) unified in the same category [14].

Our global (including childhood, adult and elderly categories) cytological series included the following distribution of diagnoses for the reference years: 13.3% for TIR1/IN, 62% for TIR2-BL (Benign Lesions-BL), 18.1% for TIR3-FN/AUS (indeterminate), 3.3% for TIR4 (Suspicious for malignancy-SM), and 3% for TIR5 (positive for malignancy-PM). All the cytological and histological sections were reviewed by 2 expert pathologists (E.D.R. and G.F.) and those cases whose interpretation was equivocal were submitted to the diagnostic judgment of the other pathologists until a final agreement was achieved. The lower limit for cytological adequacy of each sample was established according to the Bethesda and British RCPath classification scheme, which entails six groups of thyroid follicular epithelial cells within two submitted slides, and each of them, must have at least 10 well visualized viable follicular epithelial cells [14, 22].

### ICC analysis

Immunohistochemistry was run on LBC cytological specimens using the standard protocol reported in our previous papers (1-2, 20). ICC staining was performed with the avidin-biotin peroxidase complex on LBC slides using the antibodies HBME-1 (Dako, Glostrup, Denmark [1:100 dilution]) and Galectin-3 (Ventana Medical Systems, Tucson, Ariz [1:100 dilutions]. The slides were washed 3 times in phosphate-buffered saline (PBS) and then preincubated in normal veal serum with PBS at a dilution of 1:50 for 20 minutes before overnight incubation at 4°C with the primary antibody. The slides were then washed 3 times with PBS and incubated with the biotinylated secondary antibody conjugated with the avidin-biotin-peroxidase complex (Ventana Medical Systems). The reaction was developed using 3-30 diaminobenzidine as a chromogen. All slides were counterstained with hematoxylin for 5 seconds, rinsed in water 3 times, and then mounted for microscopic examination. The positivity was assessed for each cytological case when at least 50% of cells demonstrated strong cytoplasm positivity. This arbitrary 50% ICC cutoff value was established based on the histological diagnoses. The positivity of each case was defined only when a concomitant positive expression of the 2 immunomarkers was detected. Galectin-3 displayed cytoplasm staining; HBME-1 staining was within the cytoplasm with membranous and luminal accentuation. Positive controls were represented by mesothelioma cells for HBME-1 and histiocytes for galectin-3, whereas a negative control was defined by lymphocytes identified in the majority of the thyroid slides. Despite the well-known and invaluable role of cell block, our decision to apply ICC to LBC was mainly based on 2 reasons: 1) our long-standing experience with ICC on LBC and 2) because in our personal experience with ICC on cell blocks we reported some cases with contradictory results (false-positive or false-negative findings) that were not encountered on LBC. The validation of this immunopanel application to cytology and LBC was supported by our previous experiences with strict positive and negative controls (as reported above) but mainly by the good and informative correlation in the cyto-histological series analyzed with this specific immunopanel in literature.

### Histology

The 480 cases underwent histology based on the cytological diagnoses and among benign cytological diagnoses based on compressive symptoms. All surgical specimens were fixed in 10% buffered formaldehyde, embedded in paraffin and the 5 micron-thick microtomic sections were stained with hematoxylin-eosin. All the peri-thyroid adipose tissue was embedded and examined for lymph-nodes research. The diagnosis of PTC was based on the presence of true papillary structures and the distinctive nuclear features whereas the diagnosis of FVPC relied upon the detection of the nuclear features of PTC in multiple foci within the tumor including both diffuse and encapsulated variants [22-23]. The diagnosis of Tall cell variant was characterized by the predominance of neoplastic cells whose height was at least three times their width and showing the classical PTC nuclear features. The diagnosis of medullary thyroid carcinoma was characterized by the predominance of neoplastic C-cells showing the classical features of medullary thyroid carcinoma and supported by the positivity for Calcitonin and CEA and negativity for Thyroglobulin. The diagnosis of oncocytic/Hurthle carcinoma was achieved in presence of either capsular or vascular invasion in an Hurthle cell tumor. Anaplastic carcinoma was characterized by presence of atypical and pleomorphic cells with high N/C ratio and severe nuclear irregularities [22-24].

All the cases were classified according to the seventh edition of the tumor-node-metastasis-based staging system recommended by the American Joint Commission on Cancer (AJCC) [23].

### Statistical analysis

Statistical analysis was performed using GraphPad-Prism 5 software (Graph Pad Software, San Diego, CA) and MedCalc version 15.11.4 (MedCalc Software, Mariakerke, Belgium). Comparison of categorical variables was performed by chi-square statistic, using the Fisher's exact test. P-values less than 0.05 were considered as statistically significant.
